# Increased psoriasis frequency in patients with familial Mediterranean fever

**DOI:** 10.1080/03009734.2017.1423425

**Published:** 2018-01-24

**Authors:** Abdulsamet Erden, Ezgi Deniz Batu, Emrah Seyhoğlu, Alper Sari, Hafize Emine Sönmez, Berkan Armagan, Selcan Demir, Emre Bilgin, Levent Kilic, Omer Karadag, Ali Akdogan, Yelda Bilginer, Ihsan Ertenli, Sedat Kiraz, Sule Apras Bilgen, Umut Kalyoncu

**Affiliations:** a Division of Rheumatology, Department of Internal Medicine, Hacettepe University Faculty of Medicine, Ankara, Turkey; b Division of Rheumatology, Department of Pediatrics, Hacettepe University Faculty of Medicine, Ankara, Turkey, and; c Department of Internal Medicine, Hacettepe University Faculty of Medicine, Ankara, Turkey

**Keywords:** Epidemiology, familial Mediterranean fever, psoriasis, rheumatology

## Abstract

**Objective:**

Familial Mediterranean fever (FMF) is a periodic fever syndrome caused by *MEFV* mutations. FMF may be associated with psoriasis in some cases. The prevalence of psoriasis in the normal Turkish population is 0.42%. We aimed to investigate the prevalence of psoriasis among FMF patients and their relatives.

**Methods:**

FMF patients followed at Hacettepe University Adult and Pediatric Rheumatology Departments between January and August 2016 were included. FMF patients/their relatives were accepted to have psoriasis if the diagnosis was made by a dermatologist.

**Results:**

A total of 351 FMF patients (177 adults; 174 children) were included. The median (min–max) age of adult and pediatric patients was 35 (19–63) and 10 (2–18) years, respectively. Thirteen (3.7%) FMF patients (11 adults, 2 children) had psoriasis. Psoriasis was more common in adult than pediatric patients (*p* = 0.02). Psoriasis was present in 22 (12.4%) of adult and 9 (5.2%) of pediatric patients’ relatives (*p* = 0.023). The frequency of psoriasis in ≥1 relatives of FMF patients was found to be 8.8%. Abdominal pain and fever were significantly higher, and arthralgia, arthritis, pleural chest pain, and pericarditis were significantly less frequent in the pediatric group than in adults (*p* < 0.05).

**Conclusion:**

Psoriasis was more common in FMF patients than in the normal population. Thus, FMF patients should be questioned and carefully examined for psoriasis lesions and psoriasis family history. Prospective multicenter studies may be important to find the incidence of psoriasis in FMF.

## Introduction

Familial Mediterranean fever (FMF) is the most common autoinflammatory disorder characterized by periodic fever and polyserositis caused by *MEFV* mutations (1). FMF has been associated with increased risks for other inflammatory diseases such as spondyloarthropathy (SpA), inflammatory bowel disease, and vasculitis such as polyarteritis nodosa (PAN), immunoglobulin A vasculitis/Henoch–Schönlein purpura (IgAV/HSP), and Behcet’s disease (2–4). Skin manifestations are not common in FMF attacks except in erysipelas-like erythema (5).

Psoriasis is a chronic inflammatory disease that affects 2%–3% of the population (6,7). The prevalence of psoriasis in the normal Turkish population is 0.42% (8). Psoriasis is a complex disease affected by genetic and environmental factors (9). *De novo* keratinocyte proliferation with abnormal epidermal differentiation is the main histopathological process in psoriasis (9), and it is thought that keratinocytes are stimulated by an active immune system, especially T lymphocytes in focal skin lesions (10).

There are case reports for the association of psoriasis with FMF (11–13), and most recently Barut et al. demonstrated an increased frequency of psoriasis in the relatives of pediatric FMF patients in a cohort study (14). The active innate immune system in FMF may be a cause for keratinocyte stimulation and co-occurrence of psoriasis.

The aim of this study was to investigate the prevalence of psoriasis among FMF patients and their relatives.

## Patients and methods

Familial Mediterranean fever patients who were consecutively referred to the Adult and Pediatric Rheumatology Outpatient clinics of Hacettepe University between January and August 2016 were enrolled. All patients fulfilled both Tel Hashomer (15) and the Turkish pediatric FMF criteria (16).

Demographic data, clinical manifestations, and *MEFV* variant analysis were documented by medical file screening and face-to-face interviews. *MEFV* gene variant analysis was performed with Sanger sequencing, and 12 variants (E148Q, P369S, F479I, M680I [G-C], M680I [G-A], I692del, M694V, M694I, K695R, V726A, A744S, R761H) were tested for in the *MEFV* gene in our Department of Medical Biology. Investigators (authors AE, LK, BA, EDB, HES) have performed the physical examinations of all patients.

The presence of psoriasis and psoriatic arthritis in patients and their relatives (first-, second-, and third-degree), was asked for. The prevalence of psoriasis in patients with FMF was compared to the prevalence of psoriasis in the general population from previous data in the literature (8).

If the psoriasis diagnosis had been made by a dermatologist, the patient was accepted as having psoriasis. If psoriasis was suspected and the patient had skin lesions, then he/she was referred to a dermatologist for further examination. Subsequently, if the diagnosis was not confirmed by a dermatologist, we did not include these patients as having psoriasis. After the interview with the patient, a phone interview (for whether the diagnosis was made by the dermatologist or not) was done with the relatives of patients who claimed that there was psoriasis in their relatives.

The prevalence of psoriasis in a population of 18,771 subjects in Havsa (western Turkey) in a study by Cakir et al., based on 17,835 study participants, was 0.42% (8). However, this study introduced the total prevalence of psoriasis including pediatric and adult patients. Thus, there is no study presenting psoriasis prevalence in adults and children separately in Turkey.

The study was approved by the ethical committee of Hacettepe University (Ethical decision no: GO 16/500-13) and was performed in accordance with the ethical standards laid down in the 1964 Declaration of Helsinki.

### Statistical analyses

Statistical analyses were performed using the SPSS software version 21. The variables were investigated using visual (histogram, probability plots) and analytic methods (Kolmogorov–Smirnov) to determine whether or not they were normally distributed. The data of descriptive analysis were expressed as the median, minimum, and maximum values. Categorical variables were compared with the chi-square test or Fisher’s exact test where appropriate. The Mann–Whitney *U* test was used to compare the non-normally distributed continuous data between two groups. Prevalence of psoriasis in our FMF patients was compared with prevalence of psoriasis in the general Turkish population by chi-square test. To compare FMF patients with the normal population, the odds ratios (OR) and 95% confidence intervals (CI) were calculated by univariate binary logistic regression models. *p* < 0.05 was considered as statistically significant.

## Results

### Study population

A total of 351 FMF patients, 177 (50.4%) adults and 174 (49.6%) children, were included in the study. The demographic and clinical characteristics of pediatric and adult patients are presented in Table 1. The median (min–max) age at symptom onset was 3 (1–14) and 12 (0–39) years in children and adults, respectively. The median age at diagnosis was 5 (1–18) years for children and 25 (2–52) years for adults. The median age of patients at the time of inclusion was 10 (2–18) and 35 (19–63) years for children and adults, respectively. The female/male ratio was higher in children than in adults (2.34 versus 1.1, respectively; *p* < 0.001).

**Table 1.** Demographic and clinical characteristics of 177 adult and 174 pediatric patients with familial Mediterranean fever (FMF).

Characteristics	Adult patients (*n* = 177)	Pediatric patients (*n* = 174)	*p* value
Gender, female, *n* (%)	124 (70.1)	93 (53.4)	0.001
Abdominal pain, *n* (%)	159 (89.8)	166 (95.4)	0.046
Fever, *n* (%)	152 (85.9)	170 (97.7)	<0.0001
Arthralgia, *n* (%)	147 (83.1)	76 (43.7)	<0.0001
Arthritis, *n* (%)	93 (52.5)	27 (15.5)	<0.0001
Pleuritic chest pain, *n* (%)	104 (58.8)	14 (8)	<0.0001
Pericarditis, *n* (%)	7 (4)	0 (0)	0.007
Amyloidosis, *n* (%)	5 (2.8)	1 (0.6)	0.215
Family history of FMF, *n* (%)	99 (55.9)	94 (54)	0.719
Parental consanguinity, *n* (%)	44 (25)	25 (14.4)	0.012
Hemodialysis history in family associated with FMF, *n* (%)	20 (11.3)	11 (6.3)	0.132
Psoriasis, *n* (%)	11 (6.2)	2 (1.1)	0.020
Psoriasis in any degree relatives, *n* (%)	22 (12.4)	9 (5.2)	0.023
Psoriasis in first-degree relatives, *n* (%)	5 (2.8)	2 (1.1)	0.44
Psoriasis in second-degree relatives, *n* (%)	5 (2.8)	6 (3.4)	0.73
Psoriasis in third-degree relatives, *n* (%)	15 (8.5)	1 (0.6)	<0.0001
Improvement of psoriasis lesions with colchicine treatment, *n* (%)	5 (45.5)	0 (0)	0.48

FMF: familial Mediterranean fever.

### FMF-related assessments

Abdominal pain and fever were more common, and arthralgia, arthritis, pleuritic chest pain, and pericarditis were less common in the pediatric patients than in adults (*p* < 0.05; Table 1). Of note, none of the pediatric patients had pericarditis during attacks. Parental consanguinity was significantly more frequent in adults than pediatric patients (*p* = 0.012). All patients received treatment with colchicine for FMF. Colchicine doses were 1–2 mg/day in adults and 0.5–1.5 mg/day in children.

### FMF and psoriasis

Thirteen (3.7%) (11 adults, 2 children) out of 351 FMF patients had psoriasis. The prevalence of psoriasis in the normal Turkish population is 0.42%. The frequency of psoriasis in our FMF patients (3.7%) was higher than the frequency in the normal Turkish population (*p* < 0.0001). Psoriasis was more common in adults than in children (6.2% versus 1.1%, respectively; *p* = 0.02). The frequency of psoriasis in the relatives of the FMF patients was 8.8%. Psoriasis was more common in the relatives of adult than pediatric FMF patients (12.4% versus 5.2%, respectively; *p* = 0.023). In 5 (45.5%) adult patients with psoriasis, psoriatic lesions diminished with colchicine. Arthralgia, arthritis, pleuritic chest pain, and pericarditis were more common in patients with psoriasis than in patients without psoriasis (*p* < 0.05; Table 2).

**Table 2.** Comparing demographic and clinical characteristics of familial Mediterranean fever (FMF) patients with or without psoriasis.

Characteristics	Patients with psoriasis (*n* = 13)	Patients without psoriasis (*n* = 338)	*p* value
Gender, female, *n* (%)	9 (69.2)	208 (61.5)	0.5
Abdominal pain, *n* (%)	12 (92.3)	313 (92.6)	0.9
Fever, *n* (%)	12 (92.3)	310 (91.7)	0.9
Arthralgia, *n* (%)	12 (92.3)	211 (62.4)	0.028
Arthritis, *n* (%)	8 (61.5)	112 (33.1)	0.034
Pleuritic chest pain, *n* (%)	8 (61.5)	110 (32.5)	0.03
Pericarditis, *n* (%)	2 (15.4)	5 (1.5)	0.025
Amyloidosis, *n* (%)	1 (7.7)	5 (1.5)	0.204
Family history of FMF, *n* (%)	7 (53.8)	186 (55)	0.93
Parental consanguinity, *n* (%)	1 (7.7)	68 (20.2)	0.47
Hemodialysis history in family associated with FMF, *n* (%)	1 (7.7)	30 (8.9)	0.88

FMF: familial Mediterranean fever.

The *MEFV* variants in these patients (*n* = 13) were as follows: M694V/M694V in 3, M694V/- in 3, M680I/M680I in 1, M694V/M694I in 1, M694V/M680I in 1, V726A/- in 1, and E148Q in 1 patient (Table 3). Two patients had no *MEFV* analysis.

**Table 3.** The *MEFV* variants in familial Mediterranean fever (FMF) patients with and without psoriasis.

*MEFV* variants	FMF patients without psoriasis (*n* = 338) *n* (%)	FMF patients with psoriasis (*n* = 13) *n* (%)
M694V/M694V	104 (30.7)	3 (23.0)
M694V/-	43 (12.7)	3 (23.0)
M694V/M680I	32 (9.4)	1 (7.6)
M694V/V726A	24 (7.1)	0 (0)
M680I/M680I	17 (5.0)	1 (7.6)
M694V/E148Q	13 (3.8)	0 (0)
M680I/-	10 (2.9)	0 (0)
V726A/-	9 (2.6)	1 (7.6)
M680I/V726A	7 (2.0)	0 (0)
E148Q/-	7 (2.0)	1 (7.6)
M694V/R761H	5 (1.4)	0 (0)
E148Q/E148Q	4 (1.1)	0 (0)
M694V/M694I	1 (0.2)	1 (7.6)
E148Q/P369S	2 (0.5)	0 (0)
M680I/E148Q	1 (0.2)	0 (0)
M680I/R761H	1 (0.2)	0 (0)
V726A/E148Q	1 (0.2)	0 (0)
V726A/F479L	1 (0.2)	0 (0)
V726A/P369S	1 (0.2)	0 (0)
E148Q/R761H	1 (0.2)	0 (0)
P369S/P369S	1 (0.2)	0 (0)
P369S/-	1 (0.2)	0 (0)
R761H/-	1 (0.2)	0 (0)
A744S/-	1 (0.2)	0 (0)
F479L/-	1 (0.2)	0 (0)
-/-	15 (4.4)	0 (0)
*MEFV* analysis absent	34 (10.0)	2 (15.3)

MEFV: Mediterranean fever; FMF: familial Mediterranean fever.

## Discussion

Our study is the first study investigating the rates of psoriasis both in adult and pediatric FMF patients. We found that psoriasis was more common in adult than pediatric FMF patients.

When we compared the frequency of psoriasis in FMF patients with that in the normal population (8), psoriasis was more common in FMF patients (3.7% versus 0.42%, respectively; *p* < 0.0001). Adult FMF patients were more likely to have psoriasis than the normal population (OR 16.34, 95% CI 8.51–31.39; *p* < 0.0001). For pediatric FMF patients, the OR was 2.86 (95% CI 0.69–11.78; *p* = 0.21).

In a recent study, Barut et al. showed a high prevalence (20.3%) of psoriasis among family members and close relatives of patients with FMF, and only one patient had psoriasis among 202 pediatric FMF patients (14). The frequency of psoriasis in our pediatric patients was similar to this study, except that we did not find increased frequency of psoriasis in relatives of our patients. The higher frequency of psoriasis in adult than pediatric FMF patients in our study is an expected result since psoriasis peaks around 20 years of age with a second peak between 50–60 years of age (17), although it can be seen at all ages. Barut et al. reported a positive family history of psoriasis in 6% of 200 healthy Turkish controls. In our study, the family history was positive in 8.8% of the FMF patients, which was similar to their finding in the healthy controls. Of note, the frequency of positive family history for psoriasis was higher in our adult FMF patients (12.4%).

In FMF, mutant pyrin causes an increase in active interleukin-1 (IL-1) (1). In recent studies, it has been shown that IL-1 has an essential role for signaling early T helper 17 (Th17) differentiation *in vitro* and *in vivo* (18–20). Ashida et al. have shown the presence of Th17 cells in the upper dermis of psoriasis-like lesions in a patient with FMF (11). The level of IL-1 which is produced by active T lymphocytes is high in psoriasis lesions (21). It might be speculated that high IL-1 levels in FMF patients may cause Th17 activation and direct stimulation of keratinocytes; this may be the reason for the higher frequency of psoriasis in FMF patients.

Anti-inflammatory effects of colchicine, including leukocyte suppression and inhibition of cell-mediated immune responses, have made it an intriguing alternative in psoriasis treatment. The effect of colchicine in psoriasis is, however, controversial (22). No major studies have been conducted. Some studies document efficacy in many patients; others find the drug having no effect on skin lesions (23,24). In our study, 38.4% of our patients had reduction of their skin lesions under colchicine treatment.

To our knowledge, there is no study comparing clinical features of FMF patients with or without psoriasis. Interestingly, in our study arthralgia, arthritis, pleuritic chest pain, and pericarditis were more common in patients with psoriasis compared to patients without psoriasis. Close follow-up of clinical symptoms should be considered in FMF patients with psoriasis.

It is well known that there are certain differences between adult and pediatric FMF cases, such as shorter attacks in children, lack of unilateral characteristic of chest pain in some pediatric cases, more febrile attacks or even fever-only attacks in some children, and inability of some pediatric patients to express the severity and exact location of the pain (25). Regarding the characteristics of the FMF attacks, abdominal pain and fever were more common; arthralgia/arthritis, pleuritic chest pain, and pericarditis were less common in the pediatric FMF patients in our study. The reason for low frequency of pleuritic chest pain and pericarditis in pediatric patients may be that small children have difficulties in defining the ‘chest pain’ symptom. Of note, parental consanguinity was significantly more common in adults than in children with FMF; this may be explained by the decrease in consanguineous marriages in Turkey over the last 50 years (29.2% in 1968; 21.3% in 2011) (http://www.tuik.gov.tr).

Our study is limited by the confounding factors associated with any cross-sectional study; we cannot estimate the incidence of psoriasis in FMF patients. In addition, there is a lack of formal validation of self-reported medical provider diagnosis of psoriasis, especially in the relatives of FMF patients. It is also worthy of note that our study was conducted in the western part of Turkey (Havsa) and therefore may not be representative for the whole nation of Turkey. One more limitation of our study was the questioning of the patients’ relatives only by telephone interview.

In conclusion, FMF may increase the frequency of psoriasis, especially in adult patients. Mechanistic reasons for the relationships between psoriasis and FMF should be investigated in further studies. Thus, FMF patients should be questioned and carefully examined for psoriatic lesions and psoriasis family history. Further multicenter studies with long follow-up may provide more detailed incidence data for psoriasis in FMF patients.
